# Effect of Orem Self-Care Program on the Life Quality of Burn Patients Referred to Ghotb-al-Din-e-Shirazi Burn Center, Shiraz, Iran: A Randomized Controlled Trial

**Published:** 2014-01

**Authors:** Fatemeh Hashemi, Fatemeh Rahimi Dolatabad, Shahrzad Yektatalab, Mehdi Ayaz, Najaf Zare, Parisa Mansouri

**Affiliations:** 1 Department of Pediatric Nursing, Fatemeh (PBUH) School of Nursing and Midwifery, Shiraz University of Medical Sciences, Shiraz, Iran;; 2 Department of Medical-Surgical Nursing, Fatemeh (PBUH) School of Nursing and Midwifery, Shiraz University of Medical Sciences, Shiraz, Iran;; 3 Department of Mental Health & Psychiatric Nursing, Fatemeh (PBUH) School of Nursing and Midwifery, Shiraz University of Medical Sciences, Shiraz, Iran;; 4 Department of Surgery, School of Medicine, Shiraz University of Medical Sciences, Shiraz, Iran;; 5 Department of Biostatistics, School of Medicine, Infertility Research Center, Shiraz University of Medical Sciences, Shiraz, Iran

**Keywords:** Orem Self-Care, Quality of Life, Burn

## Abstract

**Background: **Advances in treatment and critical care have largely improved the survival following burns; therefore, the importance of quality of life in burn patients is an issue beyond question. The aim of this study was to determine the effects of Orem self-care program on Quality of Life of burn patients.

**Methods: **A randomized clinical trial study was conducted on 110 eligible burn patients who were selected using easy sampling method and allocated randomly into two groups of experiment and control. The instrument for data collection was a questionnaire, containing demographic and burn information and burn-specific health scale–brief (BSHS-B) questionnaire. For the experiment group, 5 sessions of theoretical training and 75-90 minutes of practical training were accomplished. The quality of life of the patients with burns was assessed in three phases by the BSHS-B questionnaire. The data were analyzed in SPSS-17 using Chi-square test, Fisher’s exact test, Independent t-test and repeated measurement multivariate test.

**Results: **After one month and two months of the use of self-care model, the quality of life of the cases improved from 73.33% to 83.78% and 98.12%, respectively (P<0.001). But the changes in the quality of life of the patients in the control group were not statistically significant (P>0.05).

**Conclusion: **Based on the obtained results of this study, designing and implementing a self-care program based on Orem’s model and the needs of burn patients will improve their quality of life. Therefore, it is recommended that this program should be considered as a part of treatment program for these patients.

**Trial Registration Number: **2013042112129N1

## Introduction

Burns and related injuries are still the major cause of mortality and disability around the world, always causing physical, psychological and economic loss in different societies; therefore, it is considered as one of the major health complications.^1^ Every year, approximately 2.4 million cases of burn injury occur in the world, 650,000 of which require treatment, 75,000 are hospitalized, and 8000-12000 die annually due to burn injuries.^2^ In the United States, it is estimated that each year about 500,000 people are treated due to burns while about 40,000 of them are hospitalized.^3^ In Iran, mortality due to burn injuries is still highly prevalent and annually about 724,000 cases occur, of whom 335,000 are cured by self treatment, 348,000 by referring to health centers and receiving gout patient services, 38,200 are admitted to a hospital, and 2920 of them die.^4^ The above statistics show that every year a number of individuals suffer from burns resulting in hospitalization, surgery, and expensive treatment.^5^ So the average annual direct and indirect costs of burn injuries are estimated to be $99,773 per patient and a large portion of this cost is spent on treatment of scars.^3^

Also, a lot of physical, psychological, social, and economic complications occurring after discharge, such as skin problems, scarring, pain, itching, stress, low self-esteem, anxiety, depression, posttraumatic stress disorder, lack of attention and support from family and friends, lack of financial support, etc., have been reported to have negative impacts on quality of life of these patients.^6^^,^^7^ Several studies have also shown that the quality of life of these patients is reduced after the incident. In a study on the quality of life of adult patients with severe burns, the findings showed that burns have negative impacts on most aspects of the patients’ quality of life including physical and mental health, and their return to work.^8^ In a study in Iran with the aim of assessing the quality of life of burn patients in Tehran, it was indicated that burn victims are faced with many problems and this incident will affect their quality of life, especially the psychological aspects.^9^ In another study with the aim of evaluating the quality of life of survivors in Pakistan, the results showed that these patients need long-term professional, physical and social support.^10^ Besides, medical treatment of burns has improved significantly in the recent decades and measuring the outcomes of burns care has changed from mortality to increase in the quality of life of burn survivors.^6^ So, today after patient’s survival, the quality of life in burn care is a priority.^11^ In recent years, the quality of life has been proposed as an important index to evaluate personal health, decide and judge the overall health of the community and find the main problems of different aspects of the individuals’ life, especially the patients with chronic conditions in medical and nursing research.^12^ Awareness about the quality of life will help the nurses as a member of the health care team to lead and improve the quality of life of the patients.^13^ Nurses have a key role in helping the burn patients to adapt to their new body image and the processes that lead to changes in their lives due to the injuries. Although care providers who work with burn patients provide the patients with information about treatment and complications of burn in the form of brochures, the initial survey and interviews with patients and their families have shown that they seriously need to learn more about burn, its complications and self-care. The required self-care and trainings in this field have been confirmed in the study of Jang et al.^3^ Although immediately after admission to an acute care unit the rehabilitation interventions begin, programs to emphasize joint movement, self-care and comprehensive counseling are essential. Moreover, the patients need to adapt to the new situation such as doing homecare, changing the lifestyle and returning to the community.^6^^,^^11^ Partridge in his study (2008) suggests that in the case of training and participation in the self-care training programs, health care will be more expensive and cause impairment in the quality of life.^14^ Promotion of self-care helps the patients to have more control over their daily lives and be more able to deal with the social performance, thereby improving their quality of life.^15^ Empowering the patients through self-care strategies improves the self-management and reduces the pain and other injuries. However, the patient will acquire enough knowledge and skill to make decisions and solve the self-related problems.^16^ One of the models that can contribute to self-care is Orem self-care model which is a nursing model and has been recommended by one of the nursing scholars named Dorothea Orem. This model focuses more on the aspects of self-care. Self-care includes activities that humans identify and carry out for their own sake in order to maintain their life and health and have constant feelings of well-being.^17^

In recent years there has been an attempt in nursing to provide care based on evidence-based research. Since the theories and models of nursing direct the clinical, research and training activities in this field, the use of nursing models is one of the key steps to achieve this goal.^18^ Orem’s Self Care Model is a suitable clinical guideline for planning and implementing the principles of self-care and is used as a conceptual framework to guide self-care programs.^19^

The Orem Self Care Model is designed in three types of care systems based on patient’s needs and conditions in health-deviation and the role of nurse: Wholly compensatory nursing system, partly compensatory nursing system, and supportive-educative nursing system. In this model when the patient is ready to learn and perform the behavior but cannot do it without help, the supportive-educative nursing system is used. Here, the nurse’s role is giving further advice. This system contributes to decision making, behavior management and acquisition of the knowledge and skills. In this study, the system for burn patients is based on the supportive- educative nursing system.^20^ Given the importance of maintaining and improving the health of patients suffering from burns and since no study on the effect of Orem self-care program on quality of life of these patients had been done, the present study was designed with the aim of considering these issues.

## Materials and Methods

This study is a randomized clinical trial in which the effect of application of Orem’s self-care program on quality of life of burn patients admitted to the burn center of Ghotb-al-Din-e-Shirazi has been investigated. A total sample size of 104 and 52 for each group with the confidence interval of 95%, test power of 90%**, **the minimum acceptable difference of 3.2 and the standard deviation of 5 was determined based on the results of the study of Zandi et al.^21^ in 2005 aiming to evaluate the effect of self-care program on the quality of life of patients with cirrhosis of the liver. Considering the attrition rate, 8 individuals were added in each group and finally 60 patients were considered for each group (total n=120) d. It is noteworthy that at the time of application, 4 patients from the experiment group and 6 from the control group were excluded due to lack of willingness to continue their participation. Therefore, the study was done on 110 subjects (56 in the experiment group and 54 in the control group). Inclusion criteria were the age range of 18-60 years, willingness to participate in the research, Persian literacy of reading and writing, no record of certified mental illness and mental retardation, and other chronic diseases (according to doctors records), access to telephone for following-up the program, having at least 10% body burns, after discharge from the hospital to complete rehab, and at last participation in all training sessions. In the case of death, incidence of stressful incident during the research (besides the burn), patients’ reluctance to continue working with the researcher for any reason and participation in any other training or counseling program parallel to the current program, the subjects were excluded from the study.

To achieve the research objectives, after the protocol was approved by the research Ethics Committee of the University with regard to moral considerations, the researcher referred to the Ghotb-al-Din-e-Shirazi and after permission from the hospital authorities, and 120 subjects were selected using easy sampling method and after giving the necessary explanation about research objectives, a written informed consent was obtained from all the participants. Then for random assignment of the subjects to the experiment and control groups, random allocation table was used. In this way, cards with numbers 1 to 120 were provided and by using random number table, all the samples were assigned to two groups. Then, again by using a random number table, one group was selected as the control group and the other group as the experiment group. 

Then an assistant researcher who was fully trained in how to complete the questionnaire and through interviews with the patient in both the experiment and control groups helped to fill out the demographic and burn information and QOL questionnaire. Then in order to design a self-care program, the researcher evaluated the patients’ needs on the basis of the literature review and by the use of the standard tool of Orem’s self-assessment. The contents of instructional sessions were designed according to the Orem’s supportive-educative nursing system and the relevant educational pamphlet was prepared. Also, the date and time of the classes were adjusted according to the experiment group’s opinion.

Instrument for data collection included demographic and burn information questionnaire, and the Burn Specific Health Scale (BSHS-B) quality of life questionnaire. In this study in order to determine the validity of the demographic and burn questionnaire, in addition to reviewing the literature and references, opinions of 10 expert professors of Shiraz Medical and Nursing School were taken into account and they were used after final approval. 

The Burn Specific Health Scale (BSHS-B) quality of life Questionnaire was developed by Kildal et al in 2001.^22^The questionnaire consists of 40 questions in physical, psychological and social areas. The physical area consists of 4 subscales of simple abilities, hand function, heat sensitivity and treatment regimens. The psychological area consists of 2 subscales of affect and body image. And finally the social area consists of 3 subscales of interpersonal relationship, sexuality and work. Every question based on the Likert scale has five options of Extremely, Quite a bit, Moderately, A little Bit and Not at all, being scored from 0-4, respectively. Thus, each question has a minimum of zero and a maximum of four scores. The maximum score of quality of life questionnaire was 160 points and the minimum score was 0. The quality of life is measured by mean scores; the greater mean is a sign of better life.

The validity of the questionnaire was confirmed in the study by Elsharbiny et al. in Egypt (2011) which was designed to investigate the quality of life of burn patients and its reliability was estimated 0.76 using Cronbach alpha coefficient for the entire questionnaire.^8^ Also in a study in Iran by Pishnamazi et al, the validity of the questionnaire was confirmed and the reliability of the whole questionnaire was estimated 0.94.^23^ Because understanding of some of the questions in the pilot study was difficult for the patients, the validity and reliability of the questionnaire was reconsidered by the researcher so that after translating the questionnaire from English to Persian, its content validity was reviewed and corrected by 10 professors of nursing and medical universities and then again by an English expert who translated it into English and confirmed it. The reliability was measured through filling out the questionnaire by 30 burn patients in Ghotb-al-Din-e-Shirazi burn clinic and the Cronbach alpha coefficient was measured to be 0.98.

The purpose of self-care is self-care of the studied units on the principles of proper nutrition and diet, proper use of medications, pain management (pharmacological and non-pharmacological), burned skin and the wound, dressing, methods to compensate for the inability to adjust temperature, exercise and physical therapy programs, uttering the changes in feelings about self-image, ways to promote coping with the changes, strengthening the ability and enhancing of self-esteem, using relaxation techniques, providing mental health and sexual relations (in married patients), which all were provided based on the literature review and the results of the survey form (assessment). 

**Figure 1:**
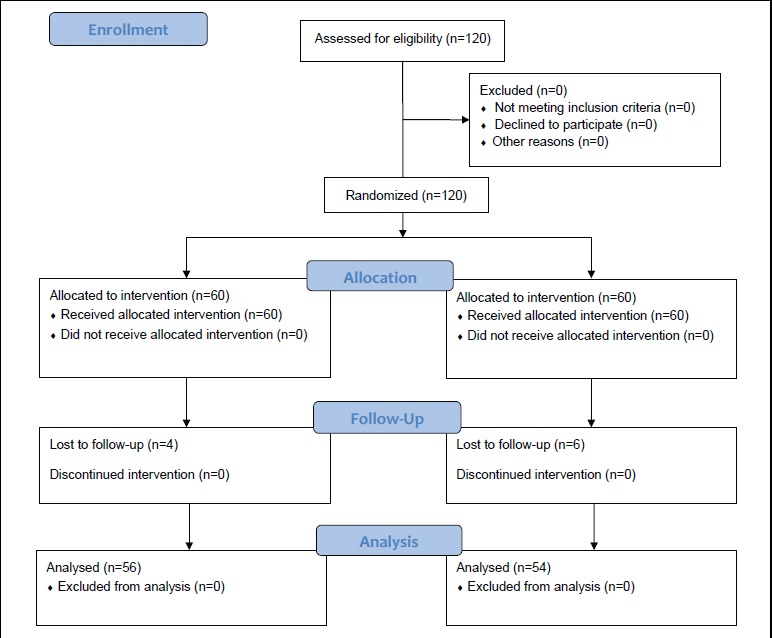
Flow diagram of subject progress through the phases of the randomized trial.

To orient the patient on the “self-care”, it is necessary to provide the necessary trainings in the mentioned areas. So, the researcher attended the clinic on Saturday to Thursday both morning and afternoon and demonstrated the self-care program that included a five-session theoretical and practical training in the form of class basis for 75 to 90 minutes of lecture, demonstration, practical implementation of health care, group discussion, cooperative learning, answering the questions, along with an attendant in groups of 3 to 5 individuals for five weeks (one session every week). The attendant was the patients’ closest family member who could involve and guide the patient in the self-care at home and could participate in all training sessions along with the patient. The attendant of the patient in this study had a supporting role and helped the patient to regain and promote independence. Each training session was prepared according to the education and understanding level of the participants and their needs by using training aids such as slides, posters and more time to justify them fully. Moreover, at the end of each session, a booklet or pamphlet and muscle relaxation training CD that contained all the points in the training sessions to refer to the brochure and pamphlet in the case of oblivion were provided for the patients and their attendants. The patients were asked to apply the compiled program for 2 months. Based on the subjects’ needs and the study objectives, necessary consultation was provided for the patients in order to gain enough knowledge and skills for self-care. In order to follow how the subjects care for themselves based on the training sessions in the previous two months, a weekly self-report checklist including the adjustments that the patient required to comply was provided for the samples. Also, at the end of each month they were checked when they referred to the center for getting a new checklist and delivering the old ones. The checklists were completed by the patients and the way to complete the self-care checklists was taught in the last session. During the two months, the researcher monitored the patients in the experiment group via phone calls and in-person consultation once every 15 days. Their questions were answered, their problems were considered and they were given advice and support for better care. In the control group, no intervention was performed after completing the quality of life questionnaire. Then, one month and two months after the end of the training, the quality of life questionnaire was filled in by the help of the researcher assistant through interviews. At the end of the intervention, in order to comply with the ethical considerations of the study, the instruction manual containing the materials provided in the training sessions was given to the control group and if they requested necessary guidance it was performed as well. Figure 1 illustrates the progress of participants through the research period. Finally the data were statistically analyzed in SPSS (version 17) and by using Chi-square test, Fisher exact test, Independent t-test and repeated measurement multivariate test in three phases.

## Results

Chi-square test, Fisher’s exact test and Independent t-test showed no significant differences between demographic and burns variables of the two groups including age, gender, marital status, education level, employment status, percentage of TBSA, length of hospital stay, time since injury, cause of burn, site of burn (tables 1 and 2).

**Table 1:** Demographic characteristics of burn patients in the two groups

**Demographic variables**	**Experiment group**	**Control group**	**P value**
Mean age (yrs) ±SD	28.05±5.69	26.74±5.78	0.233
Sex N (%)			0.960
Male	35 (62.5)	34 (63)	
Female	21 (37.5)	20 (37)	
Marital status N (%)			0.776
Single	18 (32.1)	16 (29.6)	
Married	38 (67.9)	38 (70.4)	
Education level N (%)			0.762
Reading and Writing	6 (10.7)	5 (9.3)	
Elementary	12 (21.4)	8 (14.8)	
Guidance school and diploma	30 (53.6)	35 (64.8)	
University degree	8 (14.3)	6 (11.1)	
Employment status N (%)			0.739
Housekeeper	18 (32.1)	16 (29.6)	
Worker	9 (16.1)	14 (25.9)	
Unemployed	6 (10.7)	7 (13)	
Students	3 (5.4)	3 (5.6)	
Employees & retirees	2 (3.6)	3 (5.6)	
Self-employed	18 (32.1)	11 (20.4)	

P values lower than 0.05 are statistically significant

**Table 2:** Burn data variables in the experiment and control groups

**Burn variables**	**Experiment group**	**Control group**	**P value**
Mean % of TBSA±SD	26.17±5.16	25.59±5.59	0.569
Mean Length of hospital stay(days) ±SD	20.39±12.42	19.03±11.31	0.551
Mean Time since injury(days) ±SD	75.58±37.90	82.03±55.17	0.475
Cause of burn N (%)			0.915
Thermal	44(78.6)	42(77.8)	
Chemical	6(10.7)	5(9.3)	
Electrical	6(10.7)	7(13)	
Site of burn N (%)			
Face, head & neck	31(55.4)	36(66.7)	0.224
Hand	44(78.6)	49(90.7)	0.078
Forearm(s)	28(50.0)	23(42.6)	0.436
Arm(s)	9(16.1)	13(24.1)	0.294
Trunk	24(42.9)	22(40.7)	0.822
Pelvis	9(16.1)	12(22.2)	0.412
Femur	13(23.2)	7(13)	0.163
Leg(s)	16(28.6)	18(33.3)	0.589
Foot(s)	26(46.4)	25(46.3)	0.989

P values lower than 0.05 are statistically significant

Also, data analysis before the intervention using Independent t-test has shown that the subjects’ mean of the quality of life in both experiment and control groups was not significantly different (P=0.921) and the two groups were similar in this regard before the intervention.

Table 3 shows the results of repeated measurement multivariate test based on the total score of the quality of life in burn patients in the experiment and control groups over time. According to the results of the table 3, time was a significant factor in change in the total score for quality of life (P=0.000). Regardless of time, there was a significant difference between the two groups (P=0.000). Also the difference of the interaction between time/group in both the experiment and the control groups was statistically significant (P=0.000). In this way, the experiment group showed 10.45 score increase during one month and 24.79 increase two months after the intervention compared to before the intervention while the controls showed 0.09 increase during one month and 1.43 decrease two months after the intervention compared to before the intervention. The changes were not statistically significant in the control group.

**Table 3:** Comparison of the mean change in the subjects’ quality of life in both experiment and control groups

**Variable**	**Time** ** Group **	**Before Intervention**	**1 months after intervention **	**2 months after intervention**	**Time Group Time/group**		
		**mean±SD**	**mean±SD**	**mean±SD**	**P Value**		
Quality of Life	Experiment	73.33±20.75	83.78±17.51	98.12±16.66	0.000*	0.000*	0.000*
	Control	72.94±20.79	73.03±20.36	71.51±18.48			

P values lower than 0.05 are statistically significant

## Discussion

The results have indicated an increase in the quality of life of burn patients in the time interval of before to two-months after the interventions. This shows the impact of intervention on the quality of life of the experiment group, while no significant changes were observed in the quality of life of the controls. Incidence of physical and psychosocial problems due to burn will affect the quality of life of these patients. Therefore, the more the problems are, the lower the quality of life will be. As Elsherbiny et al. confirmed in their study, burn patients have a lower quality of life.^8^ Also Grisbrook et al. in a study on 9 burn subjects showed that burned ones in comparison with their non-burned counterparts have a lower quality of life.^24^ The current study has shown that the overall mean score of quality of life of the subjects was 73.14 (73.33 in the experiment group and 72.94 in the control group) while Kvannli et al. presented the minimum score of 146-160 as improvement in the health-related quality of life after burn.^25^

The obtained results from the control group have shown a reduction trend in the quality of life of these groups in the last two months. Although the reduction was not significant, it was considerable. This reduction can largely be due to various complications such as scar, deformation, dysfunction etc. In addition to the costs, it often impairs the body image, loss of physical function followed by anxiety and depression in these patients. Moreover, in addition to improvement in the treatment of these patients, there are still some problems such as pain, itching, skin problems, lack of attention and support from family and friends, lack of financial support, etc. All of these have a negative impact on the patients’ quality of life. Therefore, measures such as educational interventions, support and empowerment should be taken to help them face the problems.

The presented results are consistent with those of some studies in this field. In a study conducted by Radwan et al. entitled “Effect of a rehabilitation program on the knowledge, physical and psychosocial functions of patients with burns“ in Egypt, it was shown that implementing a 7 day rehabilitation program for 2 weeks would improve physical, social and mental function of the patients in the experimental group.^26^ Also, the results of a study by Grisbrook et al. entitled “Exercise training to improve health related quality of life in long term survivors of major burn injury: A matched controlled study in Australia using a short questionnaire containing 36 questions showed that accomplishment of a 12-week exercise program would improve the quality of life of these patients.^24^ However, Roh etal in a study entitled “Effects of a skin rehabilitation nursing program on skin status, depression, and burn-specific health in burn survivors” in South Korea showed that the impact of this program on the quality of life of burn patients is not significant. So, the above study recommended that further studies with the use of the program should be done with more samples and in a longer duration.^27^

The results of the present study are consistent with those of other studies which confirm the positive impact of self-care program in promoting the quality of life in chronic illnesses among different age groups. In a quasi-experimental study by Asgarpour et al. in 2007 performed on 56 adolescents suffering from Hemophilia in Comprehensive Hemophilia Clinic in Tehran entitled “Effect of self-care program on quality of life in adolescents with hemophilia regarding the comprehensive hemophilia treatment center”, it was shown that the total score of the subjects’ quality of life in the experiment group compared to the control had a statistically significant difference.^28^ Also in another quasi-experimental study in 2012 performed at the Hemodialysis section of Zahedan Khatam-al-anbia Hospital entitled “Effect of applying self-care Orem model on quality of life in the patient under hemodialysis”Naroie et al showed that total score of quality of life increased significantly after the intervention.^29^ But it must be considered that their study was a quasi-experimental one with no control group and all the sessions were run individually. However, the current interventional study was designed with two experiment and control groups in order to make the conclusion more reliable. Furthermore, the training sessions were conducted in groups. The results of the present study are consistent with those of Zandi et al. and Golchin et al.; the difference was in that the quality of life of the control group significantly reduced in both studies. However both researchers had attributed it to the nature of the disease and its complications.^21^^,^^30^ In another study by Mahmodi et al entitled “Effect of self-care program on the quality of life in sickle cell anemia”, the results showed that the total score of the cases’ quality of life before and after the intervention had no significant differences while in the control group the difference was significant decreased compared to the baseline;^31^ this is inconsistent with the results of the present study. Perhaps this lack of consistency is related to the type of disease or number of training sessions, since only one training session was held in their studies while five sessions of training were held in the present study.

The results showed that designing and implementing a training program based on Orem’s theory of self-care by considering the burned patients’ needs and a follow-up can be effective in improving the quality of life of these patients. Therefore, a comprehensive, well-designed and appropriate program based on the learning needs of the client can reduce health care costs, improve quality of care, and help the clients to reduce dependency and achieve the optimal health.^31^ The obtained results of this study help the nurses to become familiar with the problems of burn patients. Therefore, medical staff, including nurses, can use the results of this study to design and train a self–care program as a part of treatment and prevent a lot of mental and psychosocial problems that can affect and reduce the patient’s quality of life. Also the obtained results on education of nursing and medical students bring about insight and awareness about the needs of these patients in order to improve their health and quality of life.

Since in the current study, due to time constraints, the effect of Orem self-care programs has been evaluated in a limited time, it is recommended that a secondary analysis of the effect of Orem self-care program in a long time (about six months after the intervention) should be done to determine to what extent these interventions are reliable. Also, by considering the inclusion criteria of this study, care should be taken in generalization of the results to people outside the research population.

## Conclusion

Generally, it can be concluded that the quality of life of burn patients who participated in the self-care program was significantly higher than that of those who did not. This result is due to the careful design and implementation of appropriate care plans tailored to the needs, interests and problems of these patients. Therefore, it is suggested that designing a self-care program based on the patients’ needs and its implementation as part of a treatment program with the aim of reducing complications and problems arising from this incident should be considered.

## References

[1] Partridge J (2005). History of Burns. N Engl J Med.

[2] Ghorbani F, Seifi B, Mohammadzade SH (2011). Microbiological factors in burn wound infection in patients hospitalized in zanjan. Nursing Research.

[3] Lo SF, Hayter M, Hsu M (2010). The effectiveness of multimedia learning education programs on knowledge, anxiety and pressure garment compliance in patients undergoing burns rehabilitation in Taiwan: an experimental study. Journal of Clinical Nursing.

[4] Rahzani K, Taleghani F, Nikbakht Nasrabadi A (2008). Qualitative Approach on Social Problems in Burned Disfigurement Individuals. Arak Medical University Journal.

[5] Ferguson SL, Voll KV (2004). Burn pain and anxiety: The use of music relaxation during rehabilitation. J Burn Care Rehabil.

[6] Liang CY, Wang HJ, Yao KP (2012). Predictors of health-care needs in discharged burn patients. Burns.

[7] Ling-Juan Z, Jie C, Jian L (2012). Development of quality of life scale in Chinese burn patients: Cross-cultural adaptation process of burn-specific health scale-brief. Burns.

[8] Elsherbiny OEE, Salem MA, El-Sabbagh AH (2011). Quality of life of adult patients with severe burns. Burns.

[9] Pishnamazy Z, Kiani-Asiabar A, Heravi-Karimooi M (2012). Quality of life in burn patients. Journal of Payesh.

[10] Tahir SM, Memon MM, Ali SA (2011). Health related Quality of life after burns:are we really treating burns?. J Ayub Med Coll Abbottabad.

[11] Brunner LS, Suddarth DS (2010). Text book of medical-surgical nursing.

[12] Karami O, Falahat-pisheh F, Jahani-Hashemi H (2010). Quality of life in cancer patients in Qazvin 2007. Journal of Qazvin University of Medical Sciences.

[13] Heravi-Karimooi M, Pourdehghan M, Faghihzadeh S (2006). The effects of group counseling on symptom scale of Quality of life in patients with breast cancer treated by chemotherapy. Journal of Behbood.

[14] Partridge MR (2008). Patient-center asthma education in the emergency department: the case in favour. Eur Respir J.

[15] Artinian NT, Magnan M, Christian W (2002). What do patient Know about their heart failure?. Applied Nursing Research.

[16] Keshtkaran Z, Ghodsbin F, Solouki S (2010). The impact of self care Education on quality of life of those clients suffering from osteoarthritis in rehabilitation centers of shiraz university of medical science. Journal of Babol University of Medical Sciences.

[17] Rostami M, Baraz-Pordanjani SH, Farzianpour F (2009). Effect of orem self care model on ederies quality of life in health care centers of masjed solaiman in 2007-2008. Journal of Arak University of Medical Sciences.

[18] Hamedanizadeh F, Mahmoudzadeh Zarandi F, Ebadi A (2010). Effectiveness of implementation of orem self-care program on headache indices in migraineur. Kowsar Medical Journal.

[19] Cox A, Hayter M, Ruane J (2010). Alternative approaches to enhanced observations in acute inpatient mental health care: areview of the literature. J Psychiatr Ment Health Nurs.

[20] McLaughlin J, Zeeberg I (1993). Self care and multiple sclerosis: a view from two cultures. Soc Sci Med.

[21] Zandi M, Alavian SM, Memarian R (2004). Assessment of the effect of self care program on quality of life in patients with cirrhosis referred to Tehran hepatitis center in 2003. The Razi Journal of Medical Sciences.

[22] Kildal M, Andersson G, Fugl-Meyer A (2001). Development of a brief version of the burn specific health scale (BSHS-B). Journal of Trauma.

[23] Pishnamazi Z, Rejeh N, Heravi-Karimooi M (2013). Validation of the Persian version of the burn specific health scale-brief. Burns.

[24] Grisbrook TL, Reid SL, Edgar DW (2012). Exercise training to improve health related quality of life in long term survivors of major burn injury: A matched controlled study. Burns.

[25] Kvannli L, Finlay V, Edgar DW (2011). Using the burn specific health scale-brief as a measure of quality of life after burn-what score should clinicians expect?. Burns.

[26] Radwan M, Samir S, Aty OA (2011). Effect of a rehabilitation program on the knowledge, physical and psychosocial functions of patients with burns. Journal of American Science.

[27] Roh YS, Seo CH, Jang KU (2010). Effects of a skin rehabilitation nursing program on skin status, depression, and burn-specific health in Burn survivors. Rehabil Nurs.

[28] Asgarpour H, Mohammadi A, Memarian R (2007). Effect of self-care program on quality of life in adolescents with hemophilia regarding to the comprehensive hemophilia treatment center. Daneshvar Medicine.

[29] Naroie S, Naji SA, Abdeyazdan GH (2012). Effect of applying self-care orem model on quality of life in the patient under hemodialysis. Zahedan J Res Med Sci.

[30] Golchin M, Shabanloei R, Asvadi I (2008). Effects of self care program on quality of life in patients with acute leukemia receiving chemotherapy. Zahedan Journal of Research In Medical Science (Tabib Shargh).

[31] Mahmodi GR, Fayazi S, Jahani S (2012). Effect of self-care program on the quality of life in sickle cell anemia. Jundishapur Sci Med J.

